# Changes in Electromyographic Activity of the Dominant Arm Muscles during Forehand Stroke Phases in Wheelchair Tennis

**DOI:** 10.3390/s23208623

**Published:** 2023-10-21

**Authors:** Khaled Abuwarda, Abdel-Rahman Akl

**Affiliations:** 1 Department of Physical Education and Kinesiology, College of Education, Qassim University, Buraidah 51452, Saudi Arabia; k.libda@qu.edu.sa; 2 Faculty of Physical Education-Abo Qir, Alexandria University, Alexandria 21913, Egypt

**Keywords:** wearable sensors, wheelchair tennis, muscle activity, EMG, forehand strokes

## Abstract

The aim of this study was to determine the muscle activations of the dominant arm during the forehand stroke of wheelchair tennis. Five players participated in the present study (age: 32.6 ± 9.9 years; body mass: 63.8 ± 3.12 kg; height: 164.4 ± 1.7 cm). The electrical muscle activity of six dominant arm muscles was recorded using an sEMG system. A significant effect of the muscle’s activity was observed, and it was shown that the muscle activation was significantly higher in the execution phase compared to the preparation phase in the anterior deltoid and biceps brachii (34.98 ± 10.23% and 29.13 ± 8.27%, *p* < 0.001); the posterior deltoid, triceps brachii, flexor carpi radialis, and extensor carpi radialis were higher in the follow-through phase than in the execution phase (16.43 ± 11.72%, 16.96 ± 12.19%, 36.23 ± 21.47% and 19.13 ± 12.55%, *p* < 0.01). In conclusion, it was determined that the muscle activations of the dominant arm muscles demonstrate variances throughout the phases of the forehand stroke. Furthermore, the application of electromyographic analysis to the primary arm muscles has been beneficial in understanding the muscular activity of the shoulder, elbow, and wrist throughout the various phases of the forehand stroke in wheelchair tennis.

## 1. Introduction

The Paralympic Games are the most important elite sports competition for individuals with impairments [1]. Participation in wheelchair sports, such as wheelchair tennis, is becoming more and more popular, and is a fantastic opportunity for individuals with impairments to engage in physical activity [2]. Wheelchair tennis was developed in 1970, and was included in the Paralympic program in 1992 during the Barcelona Paralympic Games [2,3].

Wheelchair tennis performance is determined by the player’s attributes, including their tennis skills, talent, and level of training, as well as their wheelchair’s design and the playing conditions (e.g., court surface, indoor/outdoor) [4]. Additionally, wheelchair sports have a greater difficulty, especially tennis, which is significantly harder to play since the racket must be gripped while pushing with the hands into the hand rim of the wheelchair. The maximum speed and distance travelled during the initial three pushes were decreased by the inclusion of a racket [4,5].

Due to environmental factors such as wheelchair mechanics, social factors, physical factors, lack of transportation, inaccessible fitness facilities, user attributes, and behavior, which are so-called Newell’s task constraints, wheelchair users may have low wheelchair use confidence [6,7,8,9]. The mechanical loads applied to the upper extremities are considered an important variable in wheelchair mechanics [8,9]. To reduce these mechanical loads and optimize the match performance, the interaction between the wheelchair and athlete must be effective in the most optimal way [4,9,10,11]. Wheelchair tennis is considered the most popular adaptable racket sport, but it has a high rate of shoulder complaints [12,13,14,15], and overhead activities combined with high training loads increase the heavy strain on the shoulder and might increase the risk of overuse injuries in wheelchair tennis athletes [3,16,17].

Aben et al. [18] indicated that tennis injuries are most frequently caused by single-segment overuse, which results in significant tissue load and long-term pathological alterations. In tennis, this is especially prevalent in distal small upper extremity joints, where the elbow works as a connector in the kinetic chain, receiving energy and transmitting it to distant segments [19]. When swinging repeatedly under pressure, the elbow and wrist joints’ ability to transmit and regulate force may change, and the forearm muscles may adopt particular motor activity patterns, increasing the risk of injury.

According to Willick et al.’s [20] study, 17.9% of wheelchair tennis players obtain injuries. Therefore, wheelchair tennis has a far higher risk of injury in terms of the number of injured players, with most injuries involving the shoulder (15–72% of reported injuries), with largely muscle strains, tendinopathies, and bursitis; the hand (~20% of reported injuries)/fingers (~11% of reported injuries)/arm (~10 of reported injuries); and soft tissue, accounting for 30% of all reported injuries [2,21]. In wheelchair tennis, injuries are most commonly experienced in the dominant arm, with injuries such as traumatic rupture or attenuation [14], rotator cuff tears [3,22], and stress fractures [22].

In this context, the ability to integrate multiple data sources, evaluate movement mechanics under contextually relevant conditions, and assess an individual’s body’s physiological response to repetitive mechanical loading over time is extremely valuable in the design, implementation, and evaluation of personalized training programs [23]. The common factor among studies is that no information has been obtained on the electrical activity of the muscles during actions with the most or most important repetition, neither to improve performance nor to prevent injury; this importance is supported by previous research on the significance and variety of injuries [24,25,26].

Regardless of the importance of the performance, the risk of injury, and the examination of wheelchair tennis, there has been no research into the electrical activity of the muscles during the forehand stroke in wheelchair tennis, despite many studies investigating the muscle activity of other racket games such as table tennis [27], tennis [28,29,30,31,32,33], and squash [34], and the fact that several studies have investigated temporal and biomechanical variables such as velocity, force, power, and moments, and recommended further investigations evaluating the physiological and biomechanical characteristics of wheelchair tennis [5,35,36,37,38]. According to our understanding, only velocity, push and cycle time, power output, and sprint time were measured in the literature [2,5,35].

The forehand stroke is the most common groundstroke in tennis; it is executed with the dominant forearm fully supinated and the wrist flexed in ulnar deviation. It is a highly trained movement pattern that provides for more post-impact ball velocity than the backhand drive [14,30,39].

Therefore, knowledge of muscle activity during the forehand stroke contributes to the improvement of training techniques by providing information on overall activity, rest times, repetitions of training activities, and injury risk factors [1].

Given the importance of the investigation of muscle activities on the dominant arm regarding performance and injury prevention during wheelchair tennis, this study aims to determine the muscle activations of the dominant arm muscles during the forehand stroke, explore the muscle activations around each joint of the dominant arm, and identify the differences between forehand stroke phases. We hypothesized that the muscle activations of dominant arm muscles would all increase during the execution phase, and the muscle activation would alter within forehand stroke phases.

## 2. Materials and Methods

### 2.1. Participants

Five right-handed elite male wheelchair tennis players participated in the present study (age: 32.6 ± 9.9 years; body mass: 63.8 ± 3.12 kg; height: 164.4 ± 1.7 cm). Only five high-level participants were found for wheelchair tennis and had demonstrated tennis skills, an ability based on years of playing and demonstrating these deliveries at high-level competitions. The subjects were participating in professional wheelchair tennis competitions and had official rankings in the Egyptian tennis federation. The players’ written informed consent was obtained, and the study was authorized by the institution’s studies and research ethics committee.

### 2.2. Study Design

**Figure 1:**
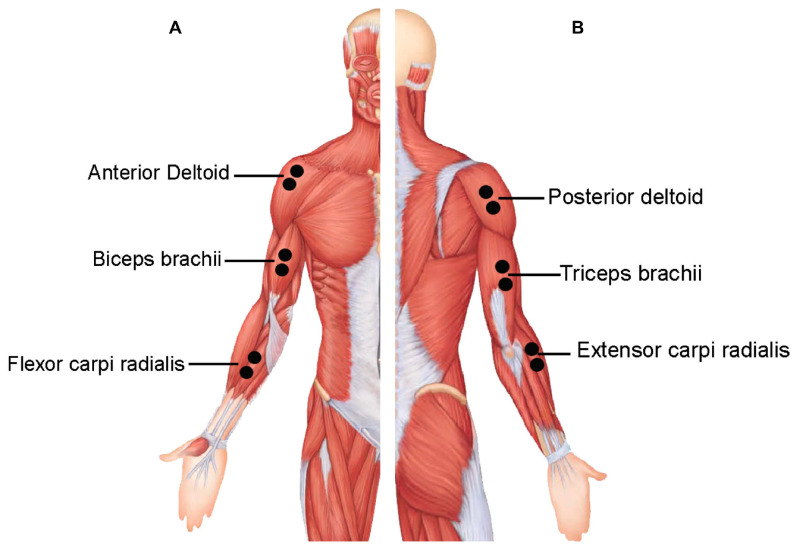
Surface electromyography (EMG) electrode placements of the selected muscles; (**A**) anterior muscles and (**B**) posterior muscles.

We used a cross-sectional design with repeated measures, in which all participants performed three successful trials as we recorded EMG measurements for the anterior deltoid, posterior deltoid, biceps brachii, triceps brachii, flexor carpi radialis, and extensor carpi radialis (Figure 1).

### 2.3. Experiment Protocol

**Figure 2:**
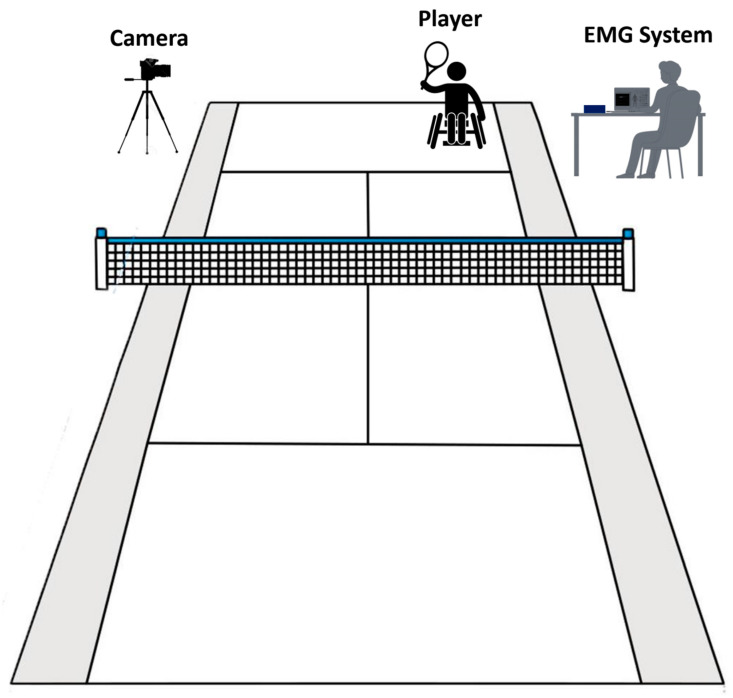
Tennis court and measurement setup.

Participants conducted the tennis forehand stroke after a 10 min warm up that included stretching and general mobility exercises for the elbows and shoulders, including WC propulsion and turning, jogging (able-bodied only), and familiarization with the protocol. Each participant had three successful tries, with a minute pause in between. The forehand stroke of wheelchair tennis was divided into three phases: the preparation phase, the execution phase, and the follow-through phase. The preparation phase was defined as the time between the start of the movement and the end of the elbow extension, the execution phase was defined as the time between the start of the elbow flexion and the shot, and the follow-through phase was defined as the time between the shot and the completion of the movement. Video recording synced with EMG was used to determine the phases. The tennis courts adhered to the standard sizes as stipulated by the International Tennis Federation. In addition, it was ensured that each participant was provided with a personal wheelchair. A standardized racket type was utilized for all the trials. Due to the unavailability of the pitching machine, all balls were served by the coach. During the trials, participants sat in a wheelchair with their feet resting on the footrests and a seatbelt fixed at the level of the anterior inferior iliac crest. The dominant hand handled the racket, while the other hand propelled the vehicle via the wheel. The athlete was urged to return the balls with the greatest possible power and steadiness, utilizing the single-handed forehand stroke to the opposite side (Figure 2) [24].

### 2.4. Data Recording

The surface EMG system (Myon m320RX; Myon, Switzerland) was utilized to record the electrical muscle activity of six dominant arm muscles. All prime mover muscles during the forehand stroke of the dominant arm were selected as the muscles most involved in wheelchair racing: the anterior deltoid, posterior deltoid, biceps brachii, triceps brachii, flexor carpi radialis, and extensor carpi radialis [21,27,29,30,32,40,41]. The skin covering the muscles of the dominant arm was shaved and cleansed with alcohol to prepare for the recording. Following that, bipolar, circular, 10 mm diameter silver chloride surface electrodes (SKINTACT FS-RG1/10, Leonhard Lang GmbH, Archenweg 56, 6020 Innsbruck, Austria) were firmly attached to the chosen muscles in accordance with SENIAM guidelines and with a 2 cm center-to-center inter-electrode spacing [42]. The EMG signals were recorded at 1000 Hz and then converted using a 16-bit analog-to-digital (A/D) converter. The EMG data were processed using Visual 3D software (C-Motion, Germantown, MD, USA). A high-pass Butterworth filter with a cut-off frequency of 25 Hz was used to reduce any artefact movement from the original EMG data. After that, the signals were rectified and low-pass filtered at 15 Hz to produce an enveloped EMG signal with a window size of 100 ms [43]. The enveloped EMG signal amplitudes were then normalized to the highest recorded signal (%MAX) throughout the three trials [42].

### 2.5. Statistical Analysis

Means and standard deviations were used to provide descriptive statistics (mean ± SD). The data distribution was examined using Shapiro–Wilk tests, and it was decided that all of the data were eligible for parametric analysis. To compare the means of each variable during the three phases, repeated measures analysis of variance (RM-ANOVA) with Sidak post hoc tests were used. The effect size was determined using partial eta squared (η2p). For all statistical analyses, IBM SPSS Statistics v27 (IBM^®^ Corporation, Armonk, NY, USA) was used.

## 3. Results

**Figure 3:**
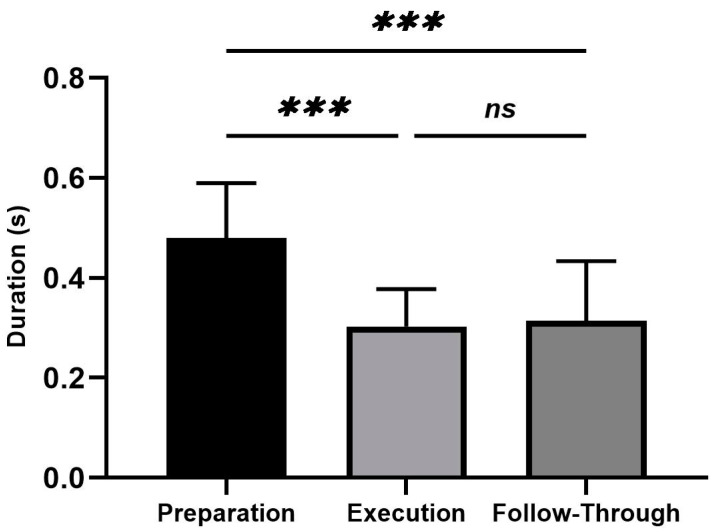
Average values and standard deviations for the duration for the forehand stroke. Significant differences for the post hoc tests between phases: (***) indicates *p* < 0.001 and (ns) indicates non-significant.

Average values and standard deviations for the duration of the forehand stroke performance are shown in Figure 3. The RM-ANOVA demonstrated significant main effects for forehand stroke during performance phases (η2p = 0.70) (Figure 3). The duration of forehand stroke performance was observed during the preparation phase, followed by the execution phase and the follow-through phase, with values of 0.48 ± 0.11 (s), 0.30 ± 0.08 (s), and 0.31 ± 0.12 (s), respectively. Additional post hoc comparisons indicated significantly less activity in the preparation compared to the execution and follow-through phases (*p* < 0.001). In addition, no significant difference was found between execution and follow-through phases (*p* = 0.968) (Figure 3).

EMG raw data (A), EMG rectified data (B), EMG RMS (C), and average values, standard deviation, and RM-ANOVA for the normalized EMG (%MAX) are presented (D) in each figure during the three analyzed phases (the preparation, the execution, and the follow-through phase).

**Figure 4:**
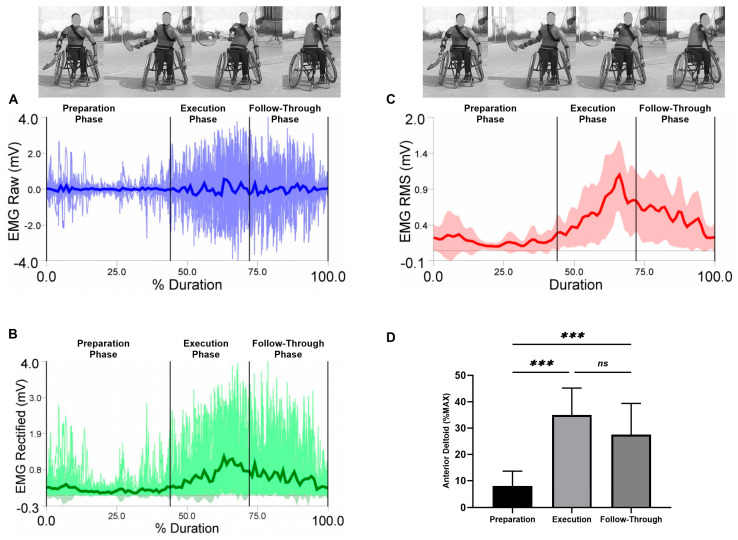
Forehand stroke phases of the anterior deltoid muscle activity. (**A**) raw data, (**B**) rectified data, and (**C**) RMS data. Stroke attempt means (solid lines) and standard deviations (shaded regions). (**D**) Mean and standard deviation of the normalized EMG (% MAX) of the anterior deltoid muscle during the preparation, execution, and follow-through phases. Significant differences for the post hoc tests between phases: (***) indicates *p* < 0.001 and (ns) indicates non-significant.

Average values and standard deviations for the selected muscles are presented in the anterior deltoid in Figure 4. The RM-ANOVA demonstrated significant main effects for forehand stroke for anterior deltoid muscle activity (η2p = 0.72) (Figure 4D). The highest activities of the anterior deltoid were observed during the execution phase, followed by the follow-through phase and the preparation phase, with values of 34.98 ± 10.23%, 27.50 ± 11.88%, and 8.00 ± 5.63%, respectively. Additional post hoc comparisons indicated significantly more activity in the preparation compared to the execution and follow-through phases (*p* < 0.001). In addition, no significant difference was found between execution and follow-through phases (*p* = 0.163) (Figure 4D).

**Figure 5:**
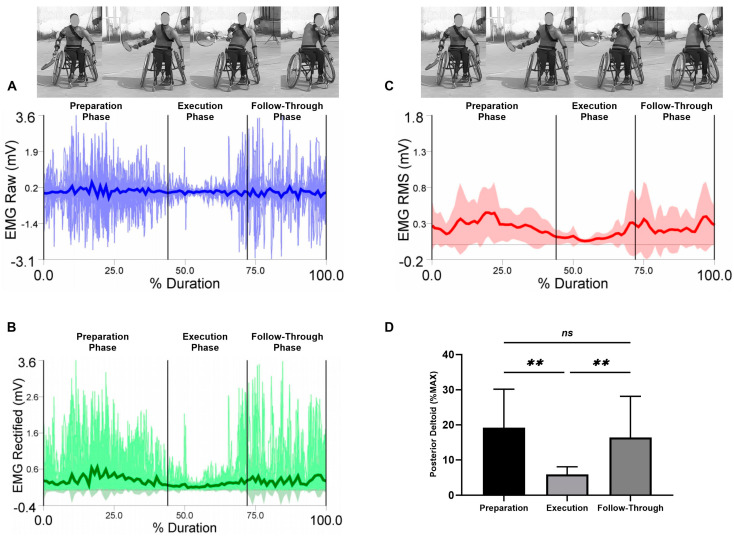
Forehand stroke phases of the posterior deltoid muscle activity. (**A**) raw data, (**B**) rectified data, and (**C**) RMS data. Stroke attempt means (solid lines) and standard deviations (shaded regions). (**D**) Mean and standard deviation of the normalized EMG (% MAX) of the anterior deltoid muscle during the preparation, execution, and follow-through phases. Significant differences for the post hoc tests between phases: (**) indicates *p* < 0.01 and (ns) indicates non-significant.

Average values and standard deviations for the selected muscles are presented for the posterior deltoid in Figure 5. The RM-ANOVA demonstrated significant main effects for forehand stroke for posterior deltoid muscle activity (η2p = 0.39) (Figure 5D). The highest activities of the posterior deltoid were observed during the preparation phase, followed by the follow-through phase and the execution phase, with values of 19.17 ± 11.02%, 16.43 ± 11.72%, and 5.88 ± 2.16%, respectively. Additional post hoc comparisons indicated significantly less activity in the preparation compared to the execution phases (*p* = 0.001). In addition, high significant increases differences of the posterior deltoid were observed between the execution and follow-through phases (*p* < 0.01). No significant difference was found between preparation and follow-through phases (*p* = 0.875) (Figure 5D).

**Figure 6:**
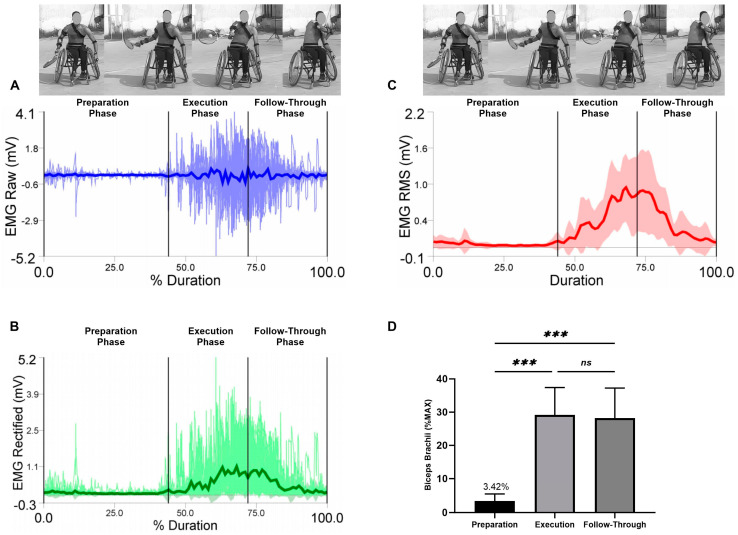
Forehand stroke phases of biceps brachii muscle activity. (**A**) raw data, (**B**) rectified data, and (**C**) RMS data. Stroke attempt means (solid lines) and standard deviations (shaded regions). (**D**) Mean and standard deviation of the normalized EMG (% MAX) of the anterior deltoid muscle during the preparation, execution, and follow-through phases. Significant differences for the post hoc tests between phases: (***) indicates *p* < 0.001 and (ns) indicates non-significant.

Average values and standard deviations for the selected muscles are presented for the biceps brachii in Figure 6. The RM-ANOVA demonstrated the most significant effects for forehand stroke for biceps brachii muscle activity (η2p = 0.78) (Figure 6D). The highest activities of the biceps brachii were observed during the execution phase, followed by the follow-through phase and the preparation phase, with values of 29.13 ± 8.27%, 28.21 ± 9.04%, and 3.42 ± 2.08%, respectively. Additional post hoc comparisons indicated significantly higher activity in the preparation compared to the execution and follow-through phases (*p* < 0.001). In addition, no significant difference was found between execution and follow-through phases (*p* = 0.994) (Figure 6D).

**Figure 7:**
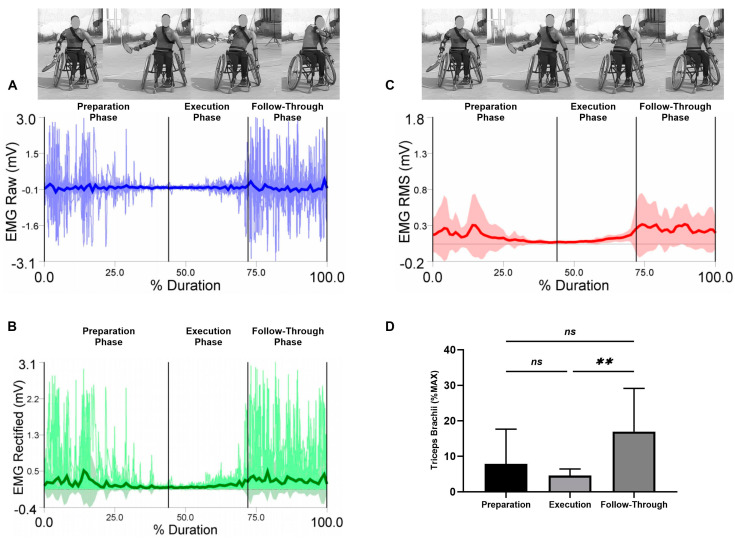
Forehand stroke phases of triceps brachii muscle activity. (**A**) raw data, (**B**) rectified data, and (**C**) RMS data. Stroke attempt means (solid lines) and standard deviations (shaded regions). (**D**) Mean and standard deviation of the normalized EMG (% MAX) of the anterior deltoid muscle during the preparation, execution, and follow-through phases. Significant differences for the post hoc tests between phases: (**) indicates *p* < 0.01 and (ns) indicates non-significant.

Average values and standard deviations for the selected muscles are presented for triceps brachii in Figure 7. The RM-ANOVA demonstrated significant main effects for forehand stroke for triceps brachii muscle activity (η2p = 0.38) (Figure 7D). The highest activities of the triceps brachii were observed during the follow-through phase, followed by the preparation phase and the execution phase, with values of 7.86 ± 9.78%, 4.53 ± 1.89%, and 16.96 ± 12.19%, respectively. Additional post hoc comparisons indicated significant increase between the execution phase compared to the follow-through phases (*p* < 0.01). In addition, no significant difference was found between preparation compared to the execution and follow-through phases (*p* = 0.543; *p* = 0.053, respectively) (Figure 7D).

**Figure 8:**
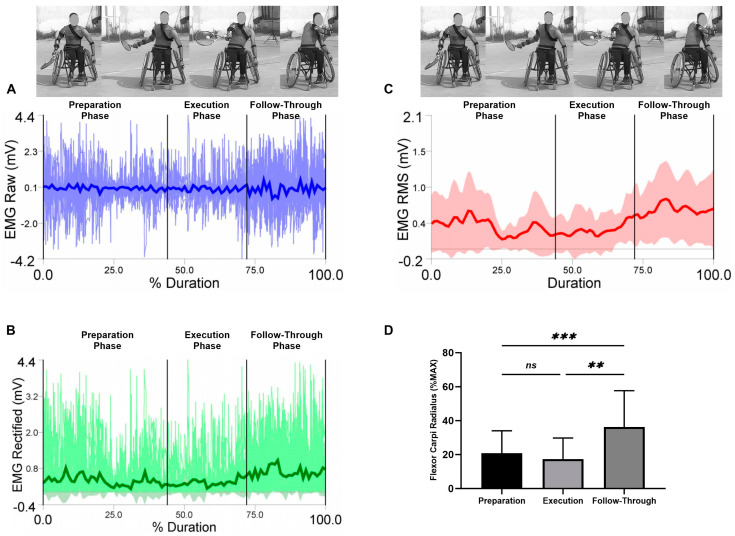
Forehand stroke phases of flexor carpi radialis muscle activity. (**A**) raw data, (**B**) rectified data, and (**C**) RMS data. Stroke attempt means (solid lines) and standard deviations (shaded regions). (**D**) Mean and standard deviation of the normalized EMG (% MAX) of the anterior deltoid muscle during the preparation, execution, and follow-through phases. Significant differences for the post hoc tests between phases: (***) indicates *p* ˂ 0.001, (**) indicates *p* ˂ 0.01 and (ns) indicates non-significant.

Average values and standard deviations for the selected muscles are presented for the flexor carpi radialis in Figure 8. The RM-ANOVA demonstrated the most significant effects for the forehand stroke for flexor carpi radialis muscle activity (η2p = 0.55) (Figure 8D). The highest activities of the flexor carpi radialis were observed during the follow-through phase, followed by the preparation phase and the execution phase, with values of 20.85 ± 13.15%, 17.37 ± 12.36%, and 36.23 ± 21.47%, respectively. Additional post hoc comparisons indicated significantly more activity in the preparation compared to the follow-through phases (*p* < 0.001). In addition, high significant differences in the flexor carpi radialis were observed between the execution and follow-through phases (*p* = 0.002). No significant difference was found between the preparation and execution phases (*p* = 0.452) (Figure 8D).

**Figure 9:**
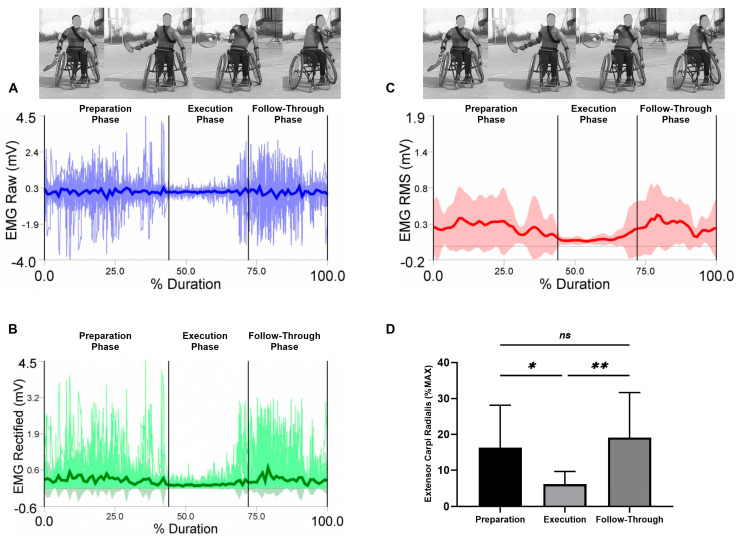
Forehand stroke phases of extensor carpi radialis muscle activity. (**A**) raw data, (**B**) rectified data, and (**C**) RMS data. Stroke attempt means (solid lines) and standard deviations (shaded regions). (**D**) Mean and standard deviation of the normalized EMG (% MAX) of the anterior deltoid muscle during the preparation, execution, and follow-through phases. Significant differences for the post hoc tests between phases: (**) indicates *p* ˂ 0.01, (*) indicates *p* ˂ 0.05 and (ns) indicates non-significant.

Average values and standard deviations for the selected muscles are presented for the extensor carpi radialis in Figure 9. The RM-ANOVA demonstrated the most significant effects for the forehand stroke for extensor carpi radialis muscle activity (η2p = 0.38) (Figure 9D). The highest activities of the extensor carpi radialis were observed during the follow-through phase, followed by the preparation phase and the execution phase, with values of 16.32 ± 11.84%, 6.11 ± 3.53%, and 19.13 ± 12.55%, respectively. Additional post hoc comparisons indicated significantly less activity in the preparation compared to the execution phases (*p* < 0.05). In addition, high significant differences in the flexor carpi radialis were observed between the execution and follow-through phases (*p* = 0.001). No significant difference was found between the preparation and follow-through phases (*p* = 0.851) (Figure 9D).

## 4. Discussion

The purpose of this study was to investigate muscle activities in the dominant arm regarding performance and injury prevention during wheelchair tennis. To our knowledge, this study is the first to provide the importance of the investigation of muscle activity during the forehand stroke using electromyographic analysis, which may help to improve training methods and quality of performance, and reduce risk factors to prevent injury.

The main activities for the anterior deltoid, posterior deltoid, biceps brachii, triceps brachii, flexor carpi radialis, and extensor carpi radialis were observed during three phases (preparation phase, execution phase, and follow-through phase), in which they acted as the prime mover muscles of the dominant arm during the forehand stroke phases in wheelchair tennis.

During the preparation phase of the shoulder joint, low levels of muscular activity were seen in the anterior deltoid muscle, where the anterior deltoid showed an activation less than 10% of MAX (8.00% MAX); the highest values of muscle activity were observed during the execution phase (34.98% MAX) and decreased during the follow-through phase to less than 30% (27.50% MAX) [30]. These results are due to the fact that the anterior deltoid muscle is an antagonist muscle during the extension and horizontal extension of the shoulder joint in the preparation phase. As a result, anterior deltoid activation was low during the first phase of the forehand stroke performance (the preparation phase). When the execution phase started, the muscle activity of the anterior deltoid increased and decreased again during the follow-through phase; these results are due to the responsibility of the anterior deltoid muscle for producing the force to flex the shoulder during the execution phase [3].

In contrast to the posterior deltoid muscle, the highest level of muscular activity was found during the preparation phase (19.17% MAX), while low values of muscle activity were observed during the execution phase (5.88% MAX), which increased during the follow-through phase (16.43% MAX). This was predicted, since the posterior deltoid muscle is an agonist muscle throughout the preparation phase of the shoulder joint’s extension and horizontal extension. Thus, the activation of the posterior deltoid was high during the first part of the forehand stroke performance (the preparation phase) and then decreased during the execution phase, in which it played the role of an antagonist muscle, and increased again during the follow-through phase. The requirement for enhanced stability during the deceleration of the forehand stroke performance might explain the simultaneous increase in muscular activity of both muscles during the follow-through phase [44]. These results provide more information about the proposed musculoskeletal adaptations and muscular activation around the shoulder, which might benefit researchers, coaches, and wheelchair tennis players in preventing shoulder injuries, which is in agreement with Mayrhuber, Rietveld, de Vries, van der Woude, de Groot, and Vegter [3].

During the preparation phase of the elbow joint, low levels of muscular activity were seen in the biceps brachii muscle during the preparation phase, where both muscles showed activations less than 10% of MAX (3.42% MAX, 7.86% MAX, respectively); the highest values of biceps brachii muscle activity were observed during the execution phase (29.13% MAX), and these values decreased during the follow-through phase (28.21% MAX), which is in agreement with Furuya, Yokoyama, Dimic, Yanai, Vogt, and Kanosue [40], and Rota, Hautier, Creveaux, Champely, Guillot, and Rogowski [41]. In contrast to the triceps brachii muscle, the highest values of muscle activity were shown during the follow-through phase (16.96% MAX), and low values of muscle activity were observed during the execution phase (4.53% MAX). This result reflects the role of muscle synergies during elbow movements, specifically during flexion [34,45,46]. Thus, the primary muscular activations of the biceps brachii and triceps brachii were identified throughout the three phases in which the elbow muscles control the position and movement of the elbow joint and stabilize the integrity of the impact-induced vibration during ball contact [47]. The triceps brachii acted as the prime mover muscle during the preparation phase, the biceps brachii acted as the main mover muscle during execution, and both muscles worked together during the follow-through phase for increased stability around the elbow joint to reduce the risk factor of injury, particularly tennis elbow, which is the most common injury in racket sports [48,49].

During the preparation phase of the wrist joint, both flexor carpi radialis and extensor carpi radialis work together during the forehand stroke phases. High values of muscle activity were shown during the preparation phase (20.85% MAX and 16.32% MAX), and activity decreased during the execution phase (17.37% MAX and 6.11% MAX), then increased during the follow-through phase (36.23% MAX and 19.13% MAX). The reason for this result may be the role of muscles around the wrist joint in the movement, not only for the forehand stroke performance but also for catching the racket, and the increase at the end of the movement range provides dynamic braking of the movement, particularly since it is known that a racket influences the performance [4,47]. Furthermore, the high muscle activations during the follow-through phase at the end of movement allow players to better prepare the arm for the following response throughout the game [45].

The muscles in the wrist possess the capability to sustain the wrist in a position that is both extended and radially offset. These muscles can counteract the force generated during acceleration and ball impact, specifically the power associated with wrist flexion [40,50]. To successfully transfer the generated proximal angular momentum distally, a rotating arm of significant length and strength must collaborate with the trunk and upper extremities [47].

This study does have some limitations that require consideration when interpreting the findings. We assumed the importance of examining the muscle co-activation, which may differ during the phases of the forehand stroke performance, and providing more information about the relationships between agonist and antagonist muscles, which our results show might be an important future research direction. The sample size is small because there are no more high-level wheelchair tennis players available. Thus, the recruitment of wheelchair tennis players, male and female, will be required for acquiring more information and identifying the differences between male and female wheelchair tennis players. Finally, the EMG data were normalized to the maximum value of the recorded signal, which is a common technique for normalizing dynamic muscle activations; however, using this technique complicates comparisons to studies that represent activation as a percentage of maximum voluntary isometric contraction (% MVIC).

Using a practical approach, the current study’s findings indicated the muscular activations of the dominant arm during the forehand stroke. Given the greater muscle activity throughout phases, we encourage wheelchair tennis coaches, therapists, and athletes to employ the current study’s muscle activity analysis to enhance neuromuscular adaptations during performance.

## 5. Conclusions

In summary, it was found that the muscle activations of the shoulder, elbow, and wrist muscles exhibit variations during the different phases of the forehand stroke in wheelchair tennis. Specifically, the anterior deltoid muscle activity demonstrated the highest values during the execution phase (34.98% MAX) and decreased during the follow-through phase to less than 30% (27.50% MAX). In contrast, the posterior deltoid muscle exhibited the highest values of muscle activity during the preparation phase (19.17% MAX), whereas low values of muscular activation were reported during the execution phase (5.88% MAX), which increased during the follow-through phase (16.43% MAX). In the muscles of the elbow, it was noted that the biceps brachii muscle exhibited the highest levels of activity during the execution phase (29.13% MAX), which subsequently decreased during the follow-through phase (28.21% MAX). Conversely, the triceps brachii muscle demonstrated the highest levels of activity during the follow-through phase, reaching 16.96% of the maximum value, while displaying low values of muscle activity during the execution phase, at only 4.53% MAX. In the context of the wrist joint, the flexor carpi radialis and extensor carpi radialis perform in concert during the forehand stroke phases. Substantial levels of muscular activity were noted during the preparatory phase (20.85% MAX and 16.32% MAX), which diminished during the execution phase (17.37% MAX and 6.11% MAX) and then increased during the follow-through phase (36.23% MAX and 19.13% MAX). Using electromyographic analysis on the primary arm muscles has been beneficial in understanding the muscular activity of the shoulder, elbow, and wrist during the phases of the forehand stroke in the context of wheelchair tennis.

## Data Availability

The data presented in this study are available on request from the Corresponding author.
